# Perfusion of Brain Preautonomic Areas in Hypertension: Compensatory Absence of Capillary Rarefaction and Protective Effects of Exercise Training

**DOI:** 10.3389/fphys.2021.773415

**Published:** 2021-12-16

**Authors:** Maria Tereza Jordão, Alexandre Ceroni, Lisete C. Michelini

**Affiliations:** ^1^Department Physiology & Biophysics, Biomedical Sciences Institute, University of São Paulo, São Paulo, Brazil; ^2^Santa Cecilia University (UNISANTA), Santos, Brazil

**Keywords:** paraventricular nucleus of the hypothalamus, nucleus of the solitary tract, capillary network, brain arteries, carotid blood flow, hemodynamic recordings, exercise training

## Abstract

Remodeling of capillary rarefaction and deleterious arteries are characteristic hallmarks of hypertension that are partially corrected by exercise training. In addition, experimental evidence showed capillary rarefaction within the brain cortex and reduced cerebral blood flow. There is no information on hypertension- and exercise-induced effects on capillary profile and function within preautonomic nuclei. We sought now to evaluate the effects of hypertension and exercise training (T) on the capillary network within hypothalamic paraventricular (PVN) and solitary tract (NTS) nuclei, and on the remodeling of brain arteries. Age-matched spontaneously hypertensive rats (SHR) and Wistar-Kyoto (WKY), submitted to moderate T or kept sedentary (S) for three months, were chronically cannulated for hemodynamic recordings at rest. Rats were anesthetized for *i.v*. administration of fluorescein isothiocyanate (FITC)-dextran (capillary volume/density measurements) or 4% paraformaldehyde perfusion (basilar, middle, and posterior arteries' morphometry) followed by brain harvesting and processing. Other groups of conscious rats had carotid blood flow (CBF, ultrasound flowmeter) acquired simultaneously with hemodynamic recordings at rest and exercise. SHR-S exhibited elevated pressure and heart rate, reduced CBF, increased wall/lumen ratio of arteries, but no capillary rarefaction within the PVN and NTS. T improved performance gain and caused resting bradycardia in both groups; reduction of pressure and sympathetic vasomotor activity and normalization of the wall/lumen ratio were only observed in SHR-T. T groups responded with marked PVN and NTS capillary angiogenesis and augmented CBF during exercise; to avoid overperfusion at rest, reduced basal CBF was observed only in WKY-T. Data indicated that the absence of SHR-S capillary rarefaction and the intense SHR-T angiogenesis within autonomic areas associated with correction of deleterious arteries' remodeling are essential adjustments to hypertension and exercise training, respectively. These adaptive responses maintain adequate baseline perfusion in SHR-S and SHR-T preautonomic nuclei, augmenting it in exercised rats when a well-coordinated autonomic control is required.

## Introduction

Accumulated experimental evidence has shown that the arteries/arterioles hypertrophy, high total peripheral resistance, increased pressure variability, capillary rarefaction, and reduced tissue perfusion are hallmarks of hypertension in both humans and experimental models of hypertension (Folkow et al., 1973; Mulvany et al., 1978; Folkow, 1982; Amaral et al., 2000; Mulvany, 2002; Melo et al., 2003; Whelton et al., 2017). Most of these studies were made in peripheral tissues such as the heart, kidney, skeletal muscle, and vasculature. Studies aiming to evaluate the effects of hypertension on brain circulatory profile were mainly directed to arteries/arterioles (Baumbach et al., 1988; Baumbach and Heistad, 1989; Sabbatini et al., 2001; Cates et al., 2012; Tayebati et al., 2012; Pires et al., 2013; De Ciuceis et al., 2018). Few studies on the density changes of hypertension-induced brain capillaries showed contradictory results, i.e., no differences between hypertensive rats and normotensive controls (Abernethy et al., 1993; Gesztelyi et al., 1993; Naessens et al., 2018) and marked hypertension-induced capillary rarefaction (Paiardi et al., 2009; Freitas et al., 2017; Plotnikov et al., 2017; Rizzoni et al., 2018). These studies were preferentially directed to cortex, hippocampus, thalamus, corpus callosum, ventromedial hypothalamus, and only two old papers analyzed the microvascular profile in brain areas responsible for the autonomic control of the circulation (Abernethy et al., 1993; Gesztelyi et al., 1993). There is no further information on the effects of hypertension-induced capillary density changes and function within the nucleus of the solitary tract (NTS) and the paraventricular nucleus of the hypothalamus (PVN), important nuclei for autonomic control. The NTS conveys the afferent drive from all peripheral receptors to brain integrative areas and the PVN is essential for the integration of autonomic, endocrine, and behavioral responses triggered by exercise. To analyze the effects of hypertension on NTS and PVN capillary network is one of the aims of the present study.

If the outcome of hypertension on the capillary network and local perfusion within preautonomic nuclei remains unclear, nothing is known about the effects of exercise training in hypertensive individuals. Experimental evidence has shown that aerobic training and antihypertensive treatments are effective in preventing capillary rarefaction and causing angiogenesis in both peripheral tissues and brain cortex (Amaral et al., 2000; Melo et al., 2003; Munzenmaier and Greene, 2006; Coimbra et al., 2008; Sabino et al., 2008; Egginton, 2009; Nascimento et al., 2010; Freitas et al., 2017). There are no studies documenting training-induced changes on capillary profile within the PVN and NTS, two essential areas for autonomic control of the circulation. To fill this gap of our knowledge is particularly important because, in a recent study, we observed that aerobic training reduced and normalized the increased blood-brain barrier leakage exhibited by PVN and NTS capillaries of the spontaneously hypertensive rats (SHR), an effect accompanied by the correction of autonomic dysfunction that characterizes chronic hypertension (Buttler et al., 2017).

In addition, experimental evidence indicated that hypertension-induced deleterious remodeling of brain arteries/arterioles was mitigated by antihypertensive treatments. Indeed, SHR and stroke-prone SHR treated with blockers of the renin-angiotensin system (RAS), antioxidants, calcium channel blockers exhibited improved vessels' structure (Hajdu et al., 1991; Baumbach and Heistad, 1992; Pires et al., 2010, 2013). To the best of our knowledge, there is no study in the literature analyzing the effects of exercise training on the structure and function of brain arteries in hypertensive rats. Since we showed previously that exercise training corrects the deleterious remodeling of peripheral vasculature (Melo et al., 2003; Jordão et al., 2011; Silva et al., 2015), we sought now to evaluate training-induced changes on main brain arteries and their effects on blood pressure and cerebral blood flow both at rest and during exercise.

Knowing that brain activity, oxygen consumption, and cerebral blood flow augment during mild to moderate acute bouts of exercise (Triantafyllou et al., 2020), we hypothesized that repetitive exercise sessions in hypertensive rats would induce adaptive changes in both brain arteries and capillary network of the preautonomic nuclei. Therefore, in the present study, we analyzed in SHR, the combined effect of hypertension and aerobic training on resting hemodynamic and autonomic parameters, on the basilar, posterior, and middle cerebral arteries' structure, and on the capillary profile within the PVN and NTS. Additionally, in conscious rats, we evaluated the effects of hypertension and exercise training on carotid blood flow (CBF) at resting condition and during a mild bout of exercise on the treadmill. Age matched sedentary and trained Wistar rats (WKY) were used as controls.

## Materials and Methods

### Ethical Approval, Animals, Training, and Sedentary Protocols

Surgical procedures and experimental protocols followed the Ethical Principles in Animal Research of the National Council of Animal Experimentation (CONCEA). The protocols were reviewed and approved by the Institutional Animal Care and Use Committee of the University of São Paulo (CEUA protocol 09/2019), Brazil.

Male SHR and WKY (8–10 weeks old) were housed in the Animal Facilities of the Department of Physiology and Biophysics, Biomedical Sciences Institute, on a 12:12-h light-dark cycle with free access to food and water. Rats were preselected for their ability to walk/run on a treadmill (Inbramed, KT-300, 10–15 min/day, 0.3–0.7 km/h, 0% grade for 1–2 weeks) because few rats (<5%) are unable to run. The pre-selection test avoids the inclusion of these rats in our protocols and warrant that all rats from sedentary and trained groups started the experiments in similar conditions.

Rats were submitted to a moderate-intensity training protocol (*T* = 50–60% of maximal exercise capacity, 0% grade, five days/week, 1 h/day) or kept sedentary (S) for three months (Ceroni et al., 2009; Jordão et al., 2011). Maximal exercise tests (Ceroni et al., 2009; Jordão et al., 2011) performed at weeks 0, 6, and 12, were used to determine (week 0), adjust the training intensity (week 6), and compare the efficacy of T and S protocols (week 12). Rats assigned to S protocol were handled every day. At the experimental week 12, SHR-S, SHR-T, WKY-S, and WKY-T were submitted to two different experimental protocols.

## Protocol 1: Evaluation of Hemodynamic Parameters and Brain Macro- and Microcirculation

### Chronic Catheterization and Functional Measurements

Rats were anesthetized (ketamine hydrochloride, 100 mg/kg + xylasine hydrochloride, 10 mg/kg *ip*.) for chronic catheterization of the left femoral artery and vein (Ceroni et al., 2009; Jordão et al., 2011). Rats were treated with prophylactic antibiotic (24,000 IU/kg Veterinary Pentabiotic, *sc*.) and analgesic (2 mg/kg ketoprofen, *sc*.) and allowed to recover. On the next day, with rats resting on their home cages, the arterial catheter was connected to the recording system (PowerLab, ADInstruments Bella Vista, NSW, Australia, 2,000 Hz sampling frequency); pulsatile arterial pressure (AP) and heart rate (HR) were continuously acquired in conscious for 30–40 min after the cessation of the exploratory activity. Records were made in conscious rats at least 24 h after the surgery and 27–30 h after the last section of training (Cavalleri et al., 2011). Because of the light anesthesia used, a good surgical skill, and the short time necessary for the completion of the surgery (~15 min), which reduced considerably the surgical stress, the rats recovered very well and were highly active on the next day, being able to run on the treadmill in a similar way they did before the anesthesia.

Time series of systolic AP (SAP) and pulse interval (PI) were used to analyze pressure and heart rate variabilities and their spectral components indicative of the autonomic control of the heart and vessels (Buttler et al., 2017). Briefly, power spectra densities at the frequency domain were evaluated using Fast Fourier Transform by Welch's method and Hanning windows with 50% overlap, using a customized routine (MATLAB 6.0, Mathworks, Natick MA, USA). The obtained spectra were integrated at low frequency (LF, 0.20–0.75 Hz), indicating both the vasomotor sympathetic activity (LF-SAP) and autonomic modulation of the heart (LF-PI) and at high-frequency domain (HF-PI, >0.75–3.0 Hz), an index of the cardiac vagal activity. The sympathovagal balance to the heart (LF/HF ratio) and the spontaneous baroreflex sensitivity through the αLF and αHF indexes were quantified as previously described (Laude et al., 2004).

### Analysis of Capillary Network Within Preautonomic Nuclei

After hemodynamic recordings, half of the rats of each group were anesthetized (ketamine 100 mg/kg + xylazine 10 mg/kg *ip*.) for withdrawal of 1 ml of blood through the arterial catheter. Then, 1 ml of fluorescein isothiocyanate (FITC)-dextran (FITC-dextran/2,000 kDa, Sigma-Aldrich, St. Louis, MO, USA) in 5% isotonic saline was injected at a slow rate *via* the venous catheter. Thereafter, 5–10 min were allowed for the recirculation of fluorescent marker before the decapitation of the rat. Brains were quickly removed, fixed in 4% paraformaldehyde in 0.01 M PBS for 24 h and crioprotected for 72 h as previously described (Buttler et al., 2017). Sequential brain sections (25 μm) of the whole PVN and NTS areas were obtained on the cryostat and mounted on glass slides with coverslip and Vectashield.

### Three- and Bi-dimensional Quantification of Capillaries

The slides were analyzed by a blind observer on a fluorescence microscope (Leica DMLB, Wetzlar, Germany, ×200 magnification) equipped with an ExiBlue camera (Imaging, Canada) for the localization of FITC-dextran filled capillaries within the PVN and NTS. In addition, we aimed to evaluate the capillary network within another important autonomic area, the rostral ventrolateral medulla (RVLM), but we got very few RVLM slices in the different groups, which precluded this analysis. Selected PVN and NTS fields were captured in consecutive optical focal planes (image size: 1,024 × 1,024 pixels, *Z*-axis: 50 focal planes, 0.3 μm each corresponding to 15 μm depth) to generate a composite three-dimensional image (deconvolution). The software (Image ProPlus, v7.01 3D software, Media Cybernetics, MD, USA) allowed the application of a set of filters to remove the background and increase the resolution. Capillary volume was measured in fixed areas of interest (AOI) superimposed on the image: AOI = 2,072,458, 679,193, and 594,840 μm^3^ for ventromedial (PVN*vm*), posterior (PVN*post*), and magnocellular (PVN*mg*) PVN nuclei, respectively; AOI = 754,666 μm^3^ for the 3 NTS nuclei: commissural (NTS*comm*), intermediate (NTS*int*), and medial (NTS*med*). Values were normalized to μm^3^/μm^3^.

To compare our results with published data, we measured the surface density of capillaries (a bi-dimensional approach) in the same PVN and NTS slices. AOIs were 1,32,225, 45,279, and 39,656 μm^2^ for PVN*vm*, PVN*post*, and PVN*mg*, respectively, and 50,311 μm^2^ for the 3 NTS nuclei. Capillary density was expressed as percentage area occupied by the capillaries in the AOI of each brain area. For both three- and bi-dimensional measurements, the left and right sides were quantified in both nuclei and the values averaged. For each PVN and NTS nuclei, we analyzed 12–20 brain slices/rat, 3–5 rats/group.

As a control for measurements made in preautonomic nuclei, we measured in the slides of the 4 experimental groups, the effects of hypertension and exercise training on capillary profile within the optic chiasma.

### Morphological/Morphometric Analysis of Brain Arteries

After the functional recordings, the other half of the rats of SHR-S, SHR-T, WKY-S, and WKY-T groups were deeply anesthetized (ketamine 300 mg/kg + xylazine 60 mg/kg *ip*.) and submitted, immediately after the respiratory arrest, to transcardiac perfusion with phosphate-buffered saline (PBS) followed by 4% paraformaldehyde (Cavalleri et al., 2011). Hearts were still beating when the perfusion (~100 mmHg for WKY, ~140 mmHg for SHR groups) started. Brains were harvested, dehydrated in graded ethanol concentrations, and embedded in histological paraplast; blocks were cut with a microtome (5 μm sections, Leica Cryostat CM3050, Germany). Slices containing transverse sections of the basilar (BA), posterior (PCA), and middle cerebral (MCA) arteries were mounted on glass slides and stained with H&E. Three slides with 5 semi-serial sections each (1 section every 25 μm, i.e., 15 sections/sample) were analyzed with a light microscope (Nikkon E1000, ×200 magnification). Images were acquired (video camera Nikkon ACT-1) and digitized for offline morphometric analysis (Image Pro Plus 5.1 software, Media Cybernetics, Silver Spring MD, USA). The analyses were made by a blind observer and included the determination of inner (IA, lumen) and outer areas (OA) of arteries, which were used for calculation of the inner and outer diameter (ID and OD) and radius (IR and OR), wall thickness (δ = OR – IR), wall/lumen ratio (δ/ID), and cross-sectional area (CSA = OA – IA).

## Protocol 2- Simultaneous Evaluation of CBF, AP, AND HR

### Chronic Implantation of Carotid Probe and Femoral Catheter

The CBF was measured in other conscious SHR-S, SHR-T, WKY-S, and WKY-T groups by means of a transit-time ultrasonic technique, we validated previously in conscious rats (Amaral and Michelini, 1997). At the beginning of the 10th experimental week, rats were anesthetized (Ketamine,100 mg/kg + Xylasine 10 mg/kg, *sc*.) for chronic implantation of the 1 mm flow probe (1RB, Transonic Systems Inc., Ithaca, NY, USA) around an isolated segment of the common carotid artery. Briefly, the left carotid was exposed and freed from the surrounding fat tissue to avoid obstruction of the ultrasonic signal. The probe was placed around the cleared section of the vessel; the segment containing the probe was covered with Lyostypt (Braun, Melsungen, Germany) to maintain vessel/probe alignment and to inhibit fat infiltration (Amaral and Michelini, 1997). The probe was fixed in the musculature, its cable was tunneled subcutaneously and fixed in the dorsal midscapular region. After prophylactic treatment with antibiotic and analgesic, the rats recover for 2 days. On the 3rd post-surgical day, the daily training sessions were restarted in T groups to maintain the performance gain attained (Amaral et al., 2001). At the end of 12th experimental week, rats were anesthetized again for chronic implantation of a femoral catheter as described above and recovered for 24–30 h.

### Simultaneous Recordings of AP, HR, and CBF in Conscious Rats

On the following day, the arterial catheter fastened to the transducer and the 1RB flow probe attached to the flowmeter (Transonic T206, Transonic Systems Inc., Ithaca, NY, USA) were connected to the recording system. Rats were placed on the treadmill and waited for a variable time period (~15–20 min) for the stabilization of cardiovascular parameters. Pulsatile values of CBF, AP, and HR were then continuously recorded at resting condition (30 min, basal values) and after 8–10 min of a mild bout of exercise on the treadmill (0.25 km/h for S groups and 0.45 km/h for T groups, corresponding to ~25% of the maximal velocity attained at the 12th experimental week).

### Statistical Analysis

Results are expressed as means ± SEM. To analyze the normality and homogeneity of the variance, data were submitted to the Shapiro–Wilk and Levene's tests. Data that violated the normality assumption were analyzed after being submitted to a logarithmic transformation. Differences between groups (SHR vs. WKY) and conditions (T vs. S) were compared by two-way factorial ANOVA (GraphPad Prism, version 8.3.1, CA, USA). Fischer's test was used as the *post-hoc* test. The correlation analyses were performed using Pearson's statistics. Differences were considered significant at *p* < 0.05.

## Results

### Efficacy of the Training Protocol and Effects of Exercise Training on Hemodynamic Values and Autonomic Control of Heart and Vessels

After 3 months of aerobic training, performance gain was similarly elevated in SHR-T and WKY-T (vs. respective S-controls, Table 1), but unchanged in S-groups. Direct measurement of AP and HR at rest showed that SHR-S vs. WKY-S had higher basal values of systolic, diastolic, and mean AP, pulse pressure, and HR (Table 1). Training caused resting bradycardia in both groups (−11 and −7% for SHR-T and WKY-T vs. respective S-controls) and a partial AP fall only in the SHR-T (−8 and −10% for diastolic and mean AP, respectively), without significant changes in systolic AP and pulse pressure (Table 1).

**Table 1:** Performance gain on the treadmill, hemodynamic parameters, and power spectral analysis values at rest in sedentary (S) and trained (T) Wistar-Kyoto (WKY) and spontaneously hypertensive rats (SHRs).

	**WKY-S**	**WKY-T**	**SHR-S**	**SHR-T**
Performance gain (km/h)	n = 15 −0.05 ± 0.03	n = 14 +0.64 ± 0.04^*†*^	n = 14 −0.15 ± 0.08	n = 15 +0.59 ± 0.05^*†*^
Hemodynamic parameters	*n = 14*	*n = 13*	*n = 13*	*n = 14*
SAP (mmHg)	151 ± 1	147 ± 3	204 ± 3^*^	199 ± 4^*^
DAP (mmHg)	111 ± 1	105 ± 2	149 ± 3^*^	137 ± 3^*^^†^
MAP (mmHg)	130 ± 2	126 ± 2	180 ± 2^*^	162 ± 5^*^^†^
ΔP (mmHg)	39 ± 1	43 ± 2	55 ± 3^*^	61 ± 3^*^
HR (b/min)	310 ± 5	289 ± 4^*†*^	362 ± 6^*^	323 ± 3^*^^†^
Power spectral analysis	n = 5	n = 5	n = 5	n = 6
SAP variability (mmHg^2^)	29.6 ± 8.7	24.0 ± 7.2	158.2 ± 31.6^*^	62.9 ± 13.2^*^^†^
LF-SAP (mmHg^2^)	5.5 ± 2.9	4.6 ± 1.5	28.8 ± 6.1^*^	14.7 ± 3.2^*†*^
PI variability (ms^2^)	32.8 ± 7.4	34.3 ± 9.2	35.7 ± 6.0	54.0 ± 8.3
LF/HF ratio	0.25 ± 0.05	0.27 ± 0.03	0.54 ± 0.12^*^	0.29 ± 0.03
αLF (ms/mmHg)	0.49 ± 0.15	0.91 ± 0.15	0.12 ± 0.03^*^	0.33 ± 0.06^*^
αHF (ms/mmHg)	0.78 ± 0.12	1.76 ± 0.26^*†*^	0.45 ± 0.14	0.95 ± 0.18^*^

*Values are means ± SEM. SAP, systolic arterial pressure; DAP, diastolic arterial pressure; MAP, mean arterial pressure; ΔP, pulse pressure; HR, heart rate; LF, low-frequency component; HF, high-frequency component; PI, pulse interval; αLF and αHF, spontaneous baroreflex sensitivity calculated with LF-PI and HF-PI components respectively, in relation to pressure changes. Group, condition, and interaction effects are presented in a Supplementary Material. Significances (p <0.05) are*

* *vs. respective WKY*,

† *vs. respective S rats*.

Compared with normotensive controls, SHR-S exhibited marked increases in SAP variability and LF-SAP (~5.2-fold increase for both). The LF/HF ratio that reflects the autonomic modulation of the heart was 2.2-fold elevated in SHR-S. Accordingly, the spontaneous baroreflex sensitivity was largely decreased (−76 and −42% for αLF and αHF, respectively, Table 1). In contrast, SHR-T vs. SHR-S showed significant reductions in SAP variability, LF-SAP, and LF/HF ratio (−60, −49, and −46%, respectively), confirming the protective effect of training to reduce sympathetic vasomotor activity, to improve and normalize autonomic modulation of the heart rate, thus reducing the SAP, the main stimulus for end-organ damage. Except for a significant spontaneous baroreflex sensitivity increase in WKY-T (αLF and αHF vs. WKY-S), training did not cause other autonomic changes in normotensive rats.

### Effects of Hypertension and Exercise Training on Capillary Network Within PVN and NTS Subnuclei

**Figure 1:**
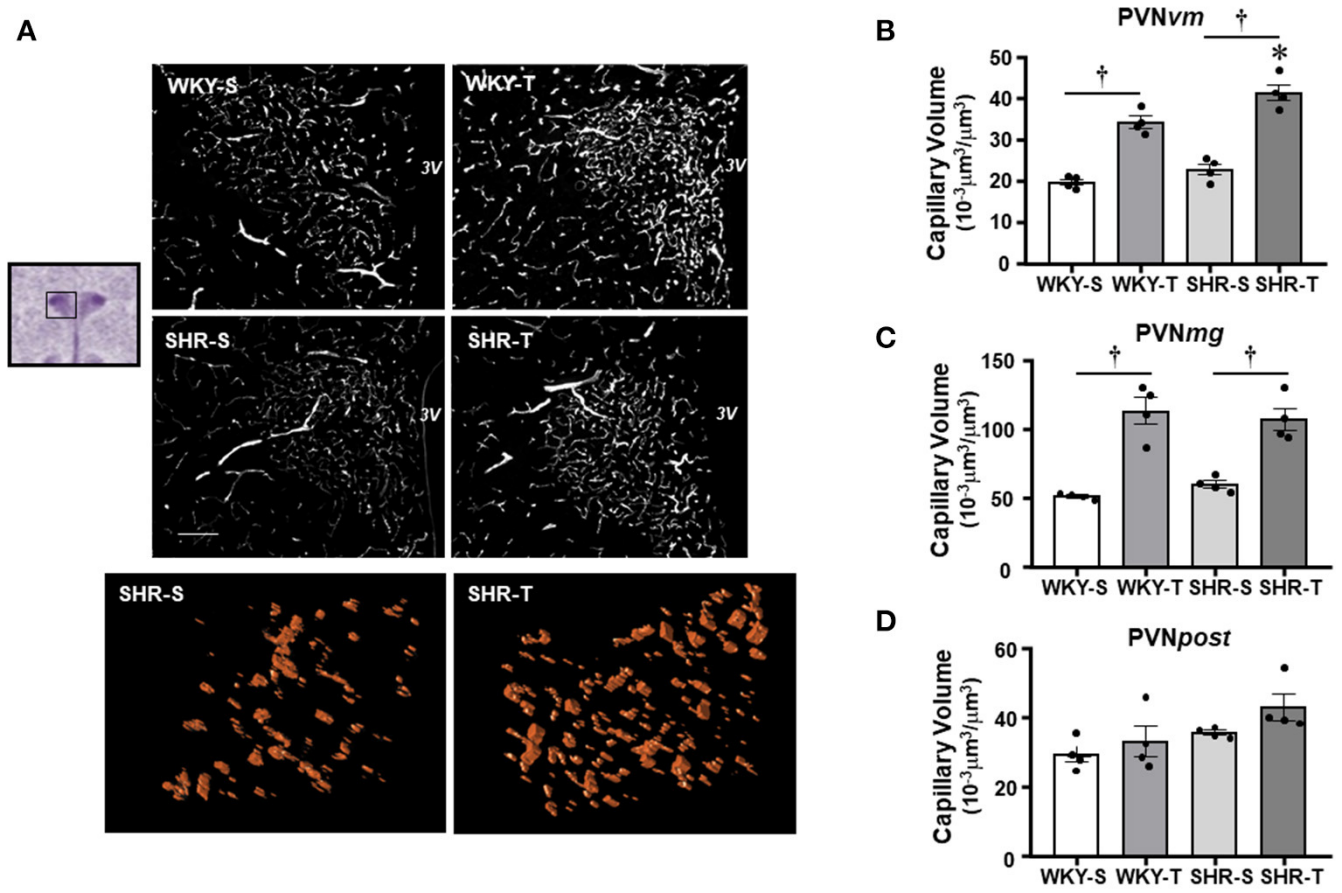
Effects of hypertension and exercise training on capillary volume within paraventricular nucleus of hypothalamus (PVN) nuclei. **(A)** Representative immunofluorescent staining of intravascular fluorescein isothiocyanate (FITC)-dextran taken from transverse sections of medial PVN of sedentary (S) and trained (T), Wistar Kyoto (WKY), and spontaneously hypertensive rat (SHR); scale bars: 50 μm; 3V, third ventricle. Enlarged drawings below are examples of the tridimensional reconstruction of the capillary profile (15 μm depth) within the ventromedial PVN (PVN*vm*) in one SHR-S (left) and other SHR-T (right). Bar graphs show the effects of hypertension and training on microcirculatory profile in the PVN*vm* **(B)**, magnocellular [PVN*mg*, **(C)**], and posterior [PVN*post*, **(D)**] nuclei of SHR and WKY submitted to S and T protocols. Measurements were made in 12–20 brain slices/rat, four rats/group. Group, condition, and interaction effects are presented in a Supplementary Material. Significances (*p* < 0.05) are: *vs. WKY, ^†^vs. S.

**Figure 2:**
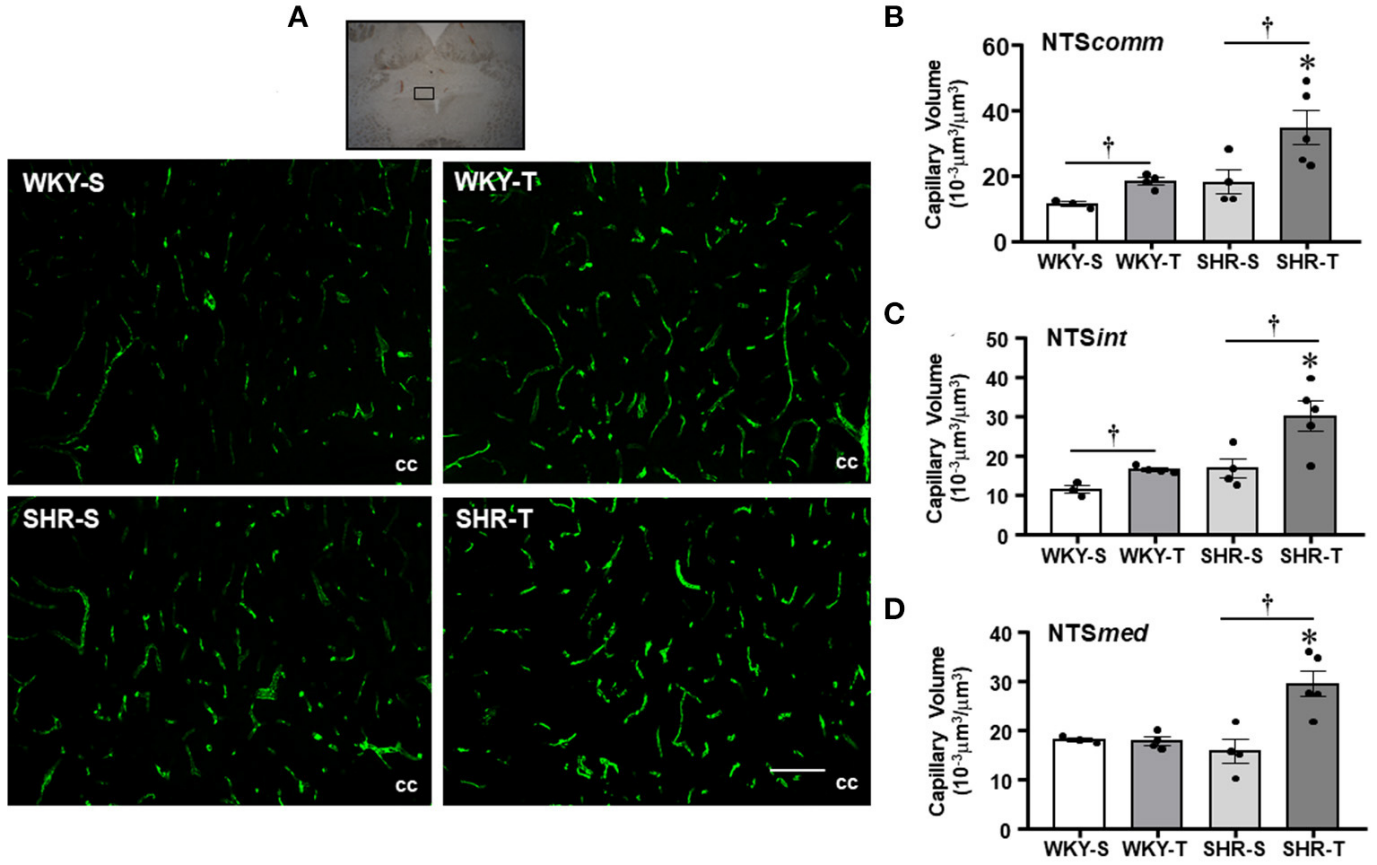
Effects of hypertension and exercise training on capillary volume within nucleus of the solitary tract (NTS) nuclei. **(A)** Representative immunofluorescent staining of intravascular FITC-dextran taken from NTS transverse sections of sedentary (S) and trained (T), WKY, and SHR; scale bars: 50 μm; cc, central canal. Bar graphs show the effects of hypertension and training on microcirculatory profile in the commissural [NTS*comm*, **(B)**], intermediate [NTS*int*, **(C)**], and medial [NTS*med*, **(D)**] nuclei of SHR and WKY submitted to S and T protocols. Measurements were made in 12–20 brain slices/rat, 3–5 rats/group. Group, condition, and interaction effects are presented in a Supplementary Material. Significances (*p* < 0.05) are: *vs. WKY, ^†^vs. S.

The effects on capillary profile were evaluated two times: by three-dimensional quantification of the capillary volume (Figures 1, 2) and confirmed by the quantitative assessment of capillary density in a surface area (bi-dimensional measurement, Table 2) as usually made.

**Table 2:** Capillary density in different subnuclei of the hypothalamic paraventricular nucleus (PVN) and nucleus of the solitary tract (NTS) in sedentary (S) and trained (T) WKY and SHRs.

	**WKY-S**	**WKY-T**	**SHR-S**	**SHR-T**
**PVN**				
ventromedial (%)	34.71 ± 1.99	53.24 ± 3.07^*†*^	41.17 ± 2.55	63.52 ± 3.97^*†*^
magnocellular (%)	89.14 ± 7.61	171.98 ± 7.17^*†*^	100.48 ± 7.89	153.84 ± 8.89^*†*^
posterior (%)	87.37 ± 4.35	97.98 ± 6.47	103.68 ± 6.37	124.25 ± 11.28
**NTS**				
commissural (%)	26.29 ± 1.30	35.25 ± 0.89^*†*^	31.96 ± 3.79	46.91 ± 5.50^*†*^
intermediate (%)	25.59 ± 1.78	33.05 ± 1.11^*†*^	32.67 ± 3.05	44.87 ± 4.25^*†*^
medial (%)	36.65 ± 2.27	35.94 ± 2.01	30.71 ± 3.14	44.04 ± 3.70^*†*^

*Values are means ± SEM and were obtained in 12–20 brain slices/rat, 3–5 rats/group. Group, condition, and interaction effects are presented in a Supplementary Material. Significances (p < 0.05) is*

† *vs. S*.

Paraventricular nucleus of hypothalamus—In WKY-S group, capillary volume and density were denser in the PVNmg and PVN*post*, showing a slightly smaller content in the PVN*vm* (Figure 1; Table 2). In contrast to our previous observation in peripheral tissues of age-matched SHR (Amaral et al., 2000; Melo et al., 2003; Coimbra et al., 2008), hypertension was not accompanied by capillary rarefaction: SHR-S capillary volume did not differ from those of the WKY-S in the 3 PVN nuclei (Figure 1). A similar observation was made for capillary density (Table 2). On the other hand, the capillary profile was significantly increased within the PVN*vm* and PVN*mg* of both trained groups (*volume changes*—SHR-T: +81% and +79%, WKY-T: +71% and +122%, respectively, Figure 1; *density changes*—SHR-T: +54% and +53%, WKY-T: +53% and +93%, respectively, Table 2). Interestingly, at the end of the training protocol, PVN*vm* volume density was even higher in the SHR-T vs. WKY-T (Figure 1). Compared with SHR-S, the SHR-T capillary network was slightly higher within PVN*post* (average increase of 20% for both volume and density), but the values did not attain significance (Figure 1; Table 2).

Nucleus of the solitary tract—The magnitude of capillary volume and density were quite similar in the different NTS subnuclei of the WKY-S. In accordance with the PVN findings, hypertension was not accompanied by capillary rarefaction within the NTS*comm*, NTS*int*, and NTS*med* (Figure 2; Table 2). Except for the NTS*med* of the WKY group, exercise training significantly increased capillary network in both strains (*volume changes*—SHR-T: +92, +78, and +87% for NTS*comm*, NTS*int*, and NTS*med*; WKY-T: +61 and +44% for NTS*comm* and NTS*int*, respectively, Figure 2; *density changes*—SHR-T: +47, +37, and +43% for NTS*comm*, NTS*int*, and NTS*med*; WKY-T: +34 and +29% for NTS*comm* and NTS*int*, respectively, Table 2). Notice that in all NTS subnuclei, capillary volume was higher in SHR-T than in age matched WKY-T submitted to the same exercise protocol.

Optic chiasm—To assess whether the effects of hypertension and training are or not specific for autonomic areas, we also quantified the capillary content within the optic chiasm. Hypertension (SHR-S vs. WKY-S) was accompanied by slight, but significant reductions in capillary volume (12.1 ± 0.9 vs. 14.4 ± 0.9 × 10^−3^ μm^3^/μm^3^, *P* < 0.05) and capillary density (18.1 ± 1.1% vs. 21.2 ± 1.2%, *P* < 0.05). Importantly, exercise training did not affect capillary content in both SHR (from 12.1 ± 0.9 to 12.9 ± 0.7 × 10^−3^ μm^3^/μm^3^ and from 18.1 ± 1.1% to 18.6 ± 1.0%, *P* > 0.05) and WKY groups (from 14.4 ± 0.9 to 15.1 ± 0.6 × 10^−3^ μm^3^/μm^3^ and from 21.2 ± 1.2% to 20.8 ± 0.9%, *P* > 0.05), as measured by three- and bi-dimensional approaches, respectively.

### Effects of Hypertension and Exercise Training on Structure of Cerebral Arteries

**Figure 3:**
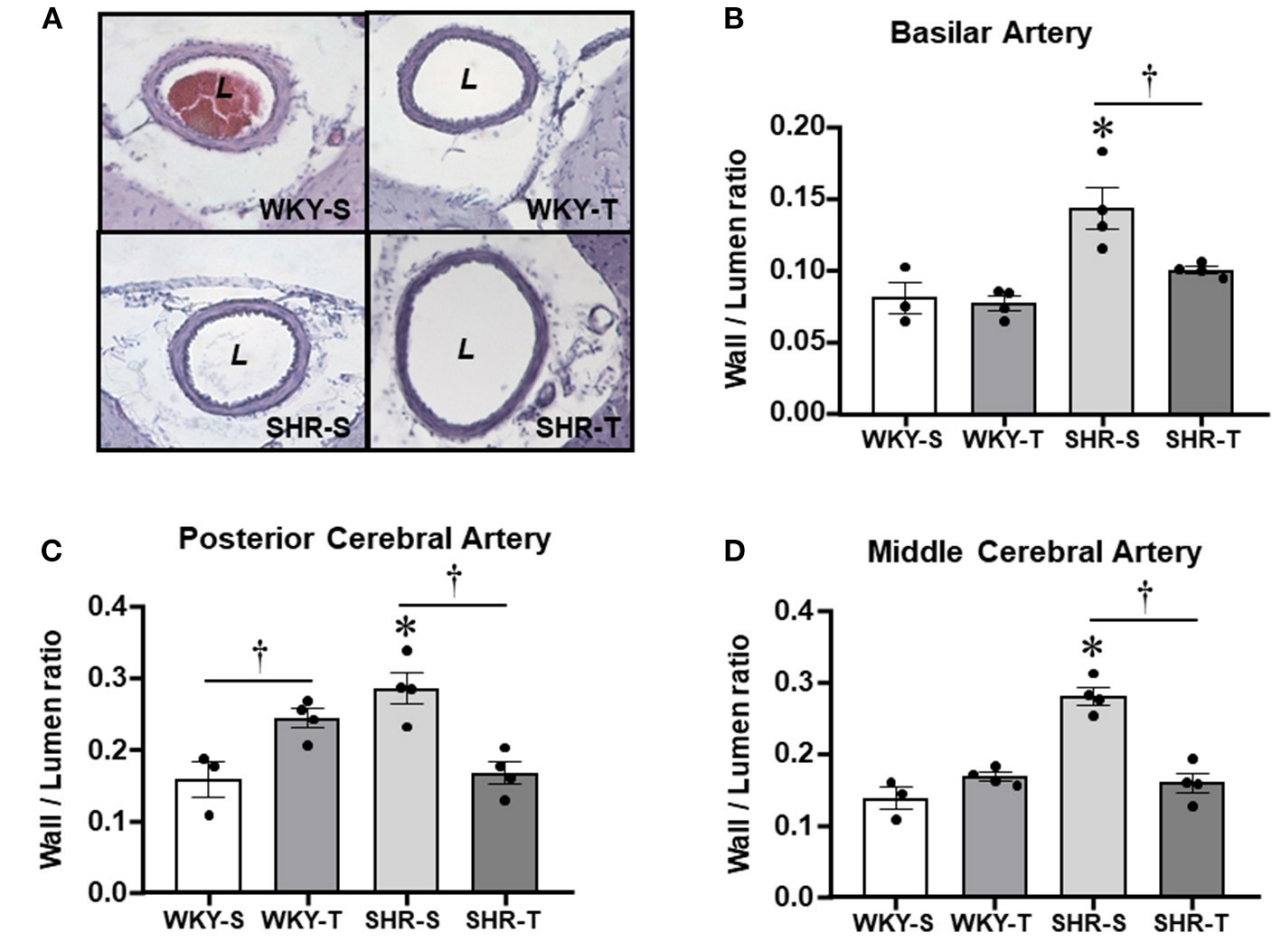
Effects of hypertension and exercise training on remodeling of brain arteries. Photomicrographs **(A)** taken from the basilar artery of sedentary (S) and trained (T), WKY and SHR; H&E staining, ×200 magnification. Bar graphs show the effects of hypertension and training on wall/lumen ratio of the basilar [BA, **(B)**], posterior [PCA, **(C)**], and middle [MCA, **(D)**] cerebral arteries of WKY and SHR groups submitted to S and T protocols. Measurements were made in 12–15 slices/rat, 3–4 rats/group. Group, condition, and interaction effects are presented in a Supplementary Material. Significances (*p* < 0.05) are *vs. WKY; ^†^vs. S.

As expected, the most prominent effect of hypertension in the cerebral arteries was the large increase in wall/lumen ratio (average of +79% in BA and PCA and +104% in the MCA for SHR-S vs. WKY-S, Figure 3). Hypertension-induced changes resulted from both the increased wall thickness (average of 22–30% in the 3 arteries) and the encroachment of lumen of arteries (ID reductions of −12, −34, and −41% in BA, PCA, and MCA, respectively, Table 3). The cross-sectional area of SHR-S arteries exhibited variable responses to hypertension: mild reduction (MCA), no change (PCA), and a slight increase (BA), characterizing eutrophic inward remodeling in MCA and PCA and hypertrophic inward remodeling in the BA (Table 3).

**Table 3:** Morphometric data in the cerebral arteries of sedentary (S) and trained (T) WKY and SHRs.

	**WKY-S**	**WKY-T**	**SHR-S**	**SHR-T**
**Basilar artery**				
ID (μm)	211 ± 9	214 ± 10	185 ± 7^*^	225 ± 7^*†*^
OD (μm)	252 ± 6	252 ± 8	236 ± 6	271 ± 8^*†*^
δ (μm)	20 ± 2	19 ± 1	26 ± 1^*^	23 ± 1^*^^†^
CSA (μm^2^)	14353 ± 645	13564 ± 398	16761 ± 347^*^	17834 ± 904^*^
**Posterior cerebral artery**				
ID (μm)	188 ± 14	160 ± 14	124 ± 5^*^	150 ± 5
OD (μm)	242 ± 14	233 ± 16	193 ± 5^*^	202 ± 6
δ (μm)	27 ± 1	36 ± 2^*†*^	35 ± 1^*^	26 ± 2^*^^†^
CSA (μm^2^)	18762 ± 1195	23920 ± 2305^*†*^	17374 ± 702	14508 ± 1159^*^
**Middle cerebral artery**				
ID (μm)	224 ± 13	164 ± 6^*†*^	132 ± 5^*^	252 ± 22^*†*^
OD (μm)	289 ± 10	220 ± 6^*†*^	210 ± 5^*^	328 ± 19^*^^†^
δ (μm)	32 ± 2	28 ± 2	39 ± 1	38 ± 3^*^
CSA (μm^2^)	29188 ± 1544	21851 ± 1395^*†*^	22701 ± 1180^*^	36467 ± 2758^*^^†^

*Values are means ± SEM and were obtained in 12–15 slices/rat, 3–4 rats/group. ID, inner diameter; OD, outer diameter; δ, wall thickness; CSA, cross-sectional area. Group, condition, and interaction effects are presented in a Supplementary Material. Significances (p < 0.05) are*

* *vs. respective WKY*,

† *vs. respective S rats*.

Although not correcting the elevated AP levels (there was only a partial decrease), exercise training reduced and normalized the wall/lumen ratio of hypertensive brain arteries (SHR-T values did not differ from those exhibited by WKY groups, Figure 3). This effect was accompanied by marked enlargement of the lumen (+22% in both BA and PCA, +91% in MCA) without (BA and PCA) or with an increase in the cross-sectional area (MCA, Table 3). Therefore, by inducing eutrophic outward remodeling (BA and PCA) and hypertrophic outward remodeling (MCA), training is largely effective in correcting the deleterious hypertension-induced remodeling. Minor geometric and wall/lumen ratio changes were observed in WKY-T vs. WKY-S (Table 3; Figure 3). The only exception was the PCA, which responded to training with a small but significant wall thickness increase for an unchanged inner diameter (Table 3), which augmented the wall/lumen ratio of WKY-T rats (Figure 3).

### Effects of Hypertension and Exercise Training on CBF at Rest and Exercise

**Figure 4:**
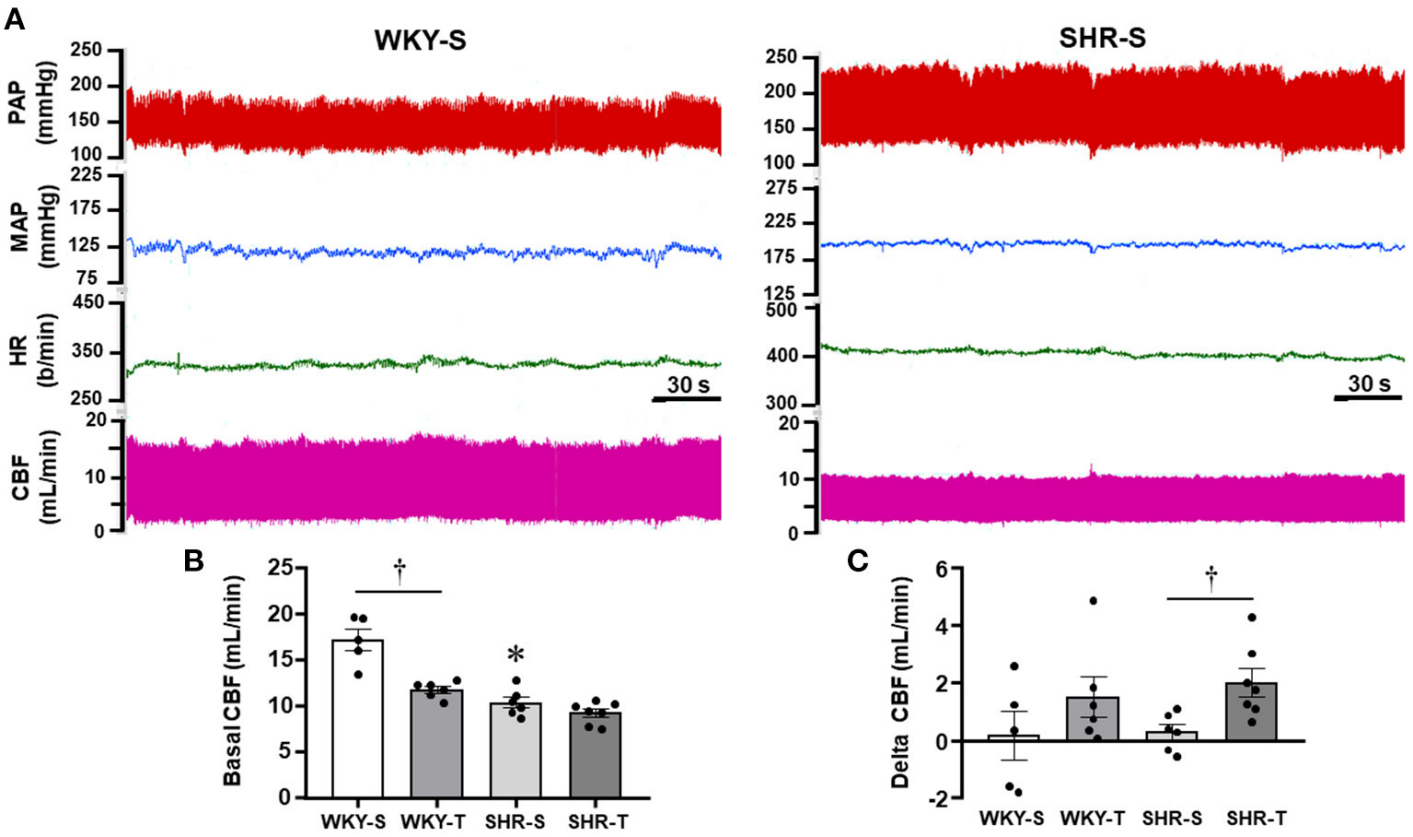
Effects of hypertension and exercise training on carotid blood flow (CBF) and hemodynamic parameters of sedentary (S) and trained (T), WKY, and SHR. **(A)** Comparison of simultaneous recordings of pulsatile (PAP) and mean arterial pressure (MAP), heart rate (HR), and pulsatile CBF at rest in one WKY-S (*left*) and one SHR-S (*right*). **(B)** Quantitative measurements of systolic CBF at basal condition. **(C)** Changes from baseline systolic CBF observed during a mild acute bout of exercise on the treadmill (Delta CBF). *n* = 5–7 rats/group. Group, condition, and interaction effects are presented in a Supplementary Material. Significances (*p* < 0.05) are: *vs. WKY, ^†^vs. S.

In other groups of conscious rats, we investigated whether hypertension- and exercise-induced adaptive vascular responses affect the CBF at rest and during an acute bout of exercise. Similar to rats used in protocol 1, performance gain was similarly elevated in SHR-T and WKY-T (+0.61 ± 0.06 and +0.60 ± 0.03 km/h) and unchanged in S groups (−0.14 ± 0.07 and −0.04 ± 0.04 km/h for SHR-S and WKY-S, respectively). Figure 4A compares simultaneous recordings of baseline values of pulsatile AP, HR, and pulsatile CBF in one SHR-S and one age-matched WKY-S. SHR-S showed higher AP (systolic, diastolic, and mean) and HR values than those of WKY-S. In contrast, pulsatile CBF was markedly reduced in SHR-S. Quantitative data indicated established hypertension and tachycardia in SHR-S (vs. WKY-S), whose values were comparable with those depicted in Table 1. Exercise-induced pressure reduction and resting bradycardia in SHR-T were like those previously observed. On the other hand, systolic values of basal CBF were 40% smaller in SHR-S compared with WKY-S (*p* < 0.05, Figure 4B). Interestingly, exercise training reduced the baseline systolic CBF in WKY-T (−31% vs. WKY-S, *p* < 0.05), with only a mild not significant change in SHR-T vs. SHR-S groups (−11%, *p* > 0,05, Figure 4B).

During the acute bouts of mild treadmill exercise, there was only mild, not significant changes on average MAP (+7 ± 5, +6 ± 4, +5 ± 4, and +6 ± 7 mmHg for WKY-s, WKY-T, SHR-S, and SHR-T, respectively). In addition, no significant CBF changes were observed in both sedentary groups (large variability with a non-significant 2% change, Figure 4C). In contrast, CBF increased in all trained rats of both groups (average increases from baseline systolic CBF were 15 and 23% in WKY-T and SHR-T, respectively, Figure 4C). Notice that during exercise bouts, the CBF was 6.7-fold higher in SHR-T compared with SHR-S (*p* < 0.05) whereas a mild not significant difference was observed between WKY-T vs. WKY-S groups (Figure 4C).

### Hypertension- and Training-Induced Changes in Baseline Pressure and CBF Correlate With Adaptive Responses in Brain Macro- and Microcirculatory Profile

**Figure 5:**
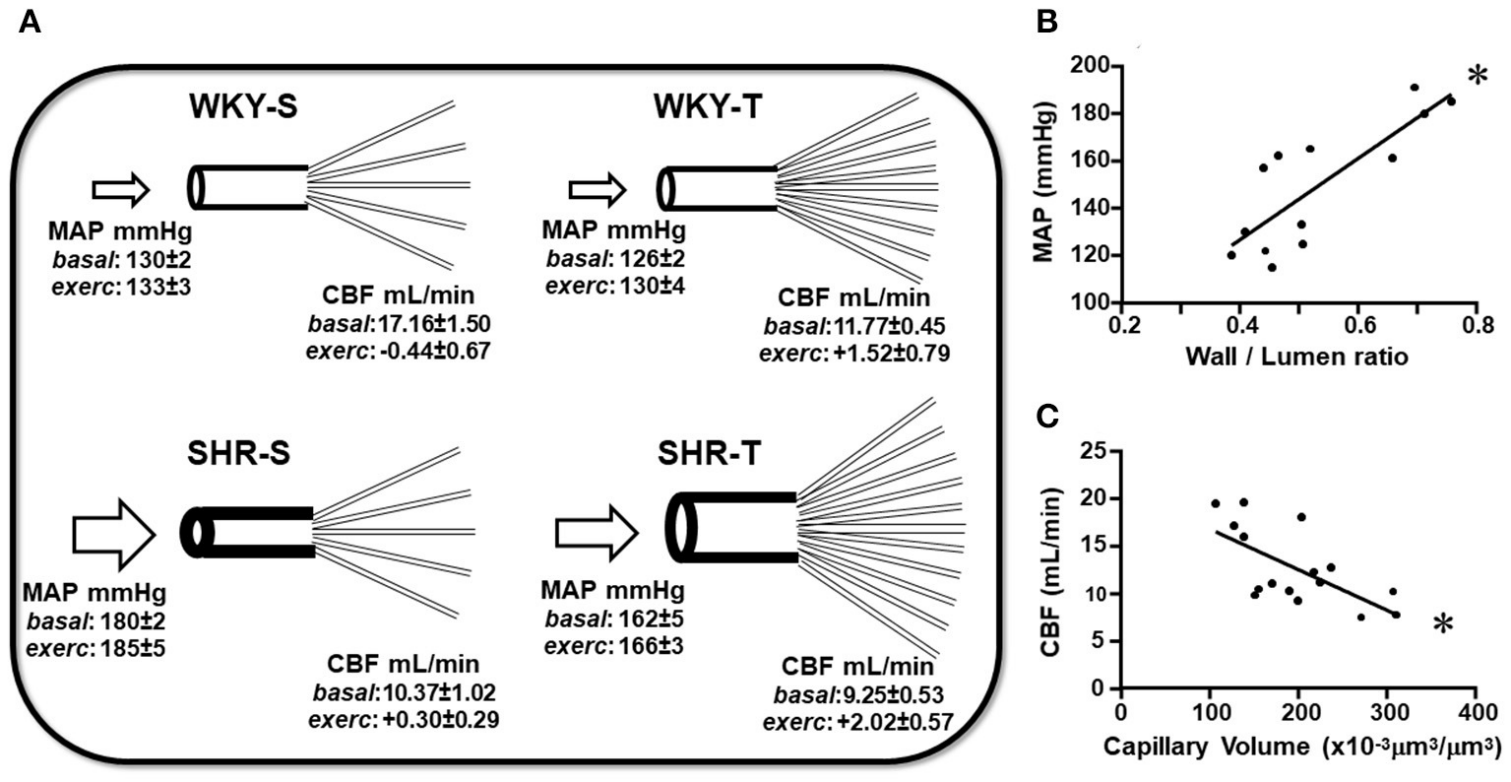
Schematic representation of the main findings of the study. **(A)** Scheme depicting the remodeling of arteries and capillary network changes induced by hypertension and exercise training in sedentary (S) and trained (T), WKY, and SHR. Mean values of MAP and CBF (at rest and exercise) for each experimental situation were presented. Graphs represent the correlations between wall/lumen ratio of cerebral arteries and MAP **(B)** and between capillary volume within preautonomic areas and CBF **(C)** in sedentary and trained normotensive and hypertensive rats. For these plots, the values of the 3 arteries and of PVN and NTS were pooled together. Wall/lumen ratio × MAP: *Y* = 172x + 58, *r* = 0.812, *p* < 0.001; Capillary Volume × CBF: *Y* = −0.043x + 21, *r* = −0.654, *p* = 0.006. *denotes a significant correlation.

Table 4 shows for the 3 arteries studied, that wall/lumen ratios were positively correlated with MAP values; on the other hand, capillary volume observed within all PVN and NTS areas was negatively correlated with baseline systolic CBF. Therefore, the high wall/lumen ratios of BA, PCA, and MCA augment the local resistance helping to maintain the elevated pressure in SHR-S; in accordance with the training-induced wall/lumen ratio reductions of the cerebral arteries contributed to MAP fall exhibited by SHR-T. Additionally, we showed that training-induced capillary angiogenesis observed in all pre-autonomic areas is essential to increase CBF during exercise (Figure 4C). At rest, however, a compensatory reduction in basal CBF (higher in WKY-T, smaller and non-significant in SHR-T vs. respective controls, Figure 4B) should occur to avoid tissue overperfusion. Figure 5 summarizes the main findings of the present study.

**Table 4:** Correlation coefficients (*r*) and *p* values of regression equations correlating wall/lumen ratio changes of cerebral arteries with baseline mean arterial pressure (MAP) and capillary volume changes within brain autonomic areas with resting carotid systolic flow (CSF).

	**Regression equation**	***r* value**	**P value**
Wall/Lumen ratio X basal MAP			
Basilar artery	Y = 635x + 83	0.776	0.002
Posterior cerebral artery	Y = 246x + 95	0.621	0.023
Middle cerebral artery	Y = 330 + 87	0.875	<0.001
Capillary volume X basal CBF			
PVN *vm*	Y = −0.29x + 21	−0.652	0.006
PVN *mg*	Y = −0.08x +19	−0.605	0.013
PVN *post*	Y = −0.29x + 23	−0.562	0.023
NTS *comm*	Y = −0.19x +16	−0.621	0.013
NTS *int*	Y = −0.27x + 17	−0.700	0.004
NTS *med*	Y = −0.32x + 18	−0.661	0.007

*Wall/lumen ratio X basal MAP correlations were made with 12–13 observations each (3–4 rats/group); those from capillary volume X basal CBF were made with 16 observations each (4 rats/group)*.

## Discussion

The present set of data confirmed both the deleterious remodeling of brain arteries and the reduced CBF in hypertension showing additional new findings, as follows: (i) there is no capillary rarefaction within the PVN and NTS subnuclei of the sedentary SHR, but a near normal capillary volume and density; (ii) aerobic training causes a marked angiogenic response within the PVN and NTS in both the SHR and WKY groups, an important adaptive response to allow increased local perfusion during exercise; (iii) training-induced augmentation in capillary network is accompanied by reduced basal CBF in WKY-T but not in SHR-T; (iv) capillary volume is negatively correlated with basal CBF in both preautonomic nuclei; (v) exercise training normalizes the wall/lumen ratio of BA, PCA, and MCA, thus contributing to decrease brain vascular resistance; and (vi) trained-induced reduction of wall/lumen ratio of arteries correlates positively with arterial pressure fall. Results uncover a compensatory adjustment within preautonomic capillary network to avoid hypoperfusion in the chronic phase of hypertension. Additionally, the data indicate the effectiveness of aerobic training to trigger angiogenesis within preautonomic areas and to oppose the deleterious hypertension-induced remodeling of brain arteries.

Accumulated experimental evidence has shown that chronic hypertension alters the structure of cerebral arteries/arterioles (increased the wall/lumen ratio caused by lumen encroachment associated with augmented wall thickness), which reduces the stress of vessels and protects downstream capillaries and venules from increased pressure levels (Baumbach et al., 1988; Baumbach and Heistad, 1989; Cates et al., 2012; Tayebati et al., 2012; Pires et al., 2013). In accordance, our morphometric analysis of BA, PCA, and MCA showed reduced lumen diameter, enlarged wall/lumen ratio, and increased resistance to blood flow in the SHR-S group when compared with age-matched WKY-S. All arteries exhibited inward remodeling, but media growth (hypertrophic remodeling) was observed only in the BA, the others showing eutrophic remodeling (Mulvany, 2002). Although the reason for this discrepancy is not apparent, Cates et al. (2012) reported that SHR exhibited higher BA wall thickness since the pre-hypertensive phase. Notice that the BA is an exception in the arterial system: 2 vertebral arteries feed into one basilar artery. To uncover the mechanism underlying BA hypertrophic remodeling, we compared WKY-S lumen diameter (data on Table 3) with that of vertebral artery measured in 3 WKY-S rats (total surface area of the 2 vertebral arteries was 370 ± 29 μm) and found a 43% lumen diameter reduction in the transition from the vertebral arteries to BA. This drastic reduction in surface area should increase the blood flow velocity, thus modifying the local remodeling. Different remodeling processes in large and pial arteries were described previously (Baumbach and Heistad, 1992; Mulvany, 2002; Kumai et al., 2008; Pires et al., 2013).

Similar to the previous findings (Grabowski et al., 1993; Tayebati et al., 2012; Pires et al., 2013; Plotnikov et al., 2017; Triantafyllou et al., 2020), we observed that chronic hypertension was accompanied by a significant reduction of basal CBF. However, in contrast to observations on cerebral cortex and retina (Paiardi et al., 2009; Freitas et al., 2017; Plotnikov et al., 2017; De Ciuceis et al., 2018; Rizzoni et al., 2018) and similar to previous reports (Abernethy et al., 1993; Gesztelyi et al., 1993; Naessens et al., 2018), the PVN and NTS of the SHR-S did not show rarefaction, but capillary volume and density values, such as those observed in WKY-S controls. Indeed, the absence of PVN capillary rarefaction was previously described in fixed post-mortem samples of 19 human subjects aged 30–85 years (Abernethy et al., 1993): no hypertension-related changes were observed, but only an aging-induced decrease in capillary profile. Grabowski et al. (1993) showed that neocortex capillary density did not differ between SHR and WKY. The absence of capillary rarefaction within the PVN and NTS seems to be a specific response of preautonomic nuclei since the optic chiasma of the SHR-S, quantified in the same slides, exhibited capillary rarefaction. Notice that although located in the same PVN slice being analyzed, vascularization of the optic chiasma could be a caveat of this study since it represents a white matter, not a gray matter vascularization.

Of importance are our findings that exercise training exhibited marked capillary angiogenesis within preautonomic areas and increased CBF during exercise in both trained groups, with a larger effect in hypertensive rats. In addition, exercise training also corrected the deleterious hypertension-induced remodeling of BA, PCA, and MCA. These changes resemble the effects induced in brain capillaries and arteries/arterioles by blockade of the RAS (Hajdu et al., 1991; Baumbach and Heistad, 1992; Saavedra et al., 2006; Kumai et al., 2008; Estato et al., 2013; Pires et al., 2013; Freitas et al., 2017; Plotnikov et al., 2017). By treating SHR with pressor and sub-pressor doses of AT1 receptor antagonist, Kumai et al. (2008) showed that both attenuated the superoxide levels in BA and MCA, reversed hypertension-induced hypertrophy and oxidative stress, and improved cerebral blood flow autoregulation, suggesting the blockade of Ang II (and its downstream pathways) as the underlying mechanism. Indeed, blood pressure fall alone is not sufficient to correct the inward remodeling of the SHR's MCA (Saavedra et al., 2006). Likewise, exercise training determined outward remodeling of BA, PCA, and MCA, with complete normalization of their wall/lumen ratios. In the SHR, cerebral cortex capillary angiogenesis induced by RAS blockade was also accompanied by marked reduction in the brain oxidative stress (Estato et al., 2013). Although studies evaluating the effects of both RAS blockade and exercise training on capillary network always exhibited a partial but significant pressure reduction (Suzuki et al., 1997; Amaral et al., 2000; Melo et al., 2003; Sabino et al., 2008; Nascimento et al., 2010; Estato et al., 2013; Freitas et al., 2017), Munzenmaier and Greene (2006) and Plotnikov et al. (2017) reported that changes in microvessel density are independent of pressure changes. We did not evaluate the participation of RAS, but it is likely that decreased Ang II availability (and the reduced activity of its downstream pathways) contributes to training-induced changes, since we showed previously downregulation of angiotensinogen and AT1 receptor expression in brain preautonomic areas and reduced both angiotensinogen expression and Ang II content within arteries of age-matched SHR submitted to a similar training protocol (Chaar et al., 2015; Silva et al., 2015, 2017; Buttler et al., 2017). Indeed, Ang II, not pressure, determined the increased capillary leakage within autonomic nuclei of the SHR-S (Biancardi et al., 2014; Buttler et al., 2017) and training-induced RAS downregulation was the main factor for correcting the capillary leakage in the trained SHR (Buttler et al., 2017). The interplay among intravascular pressure, angiotensin II, and other humoral agents, as well as neural and genetic factors on exercise-induced arteries outward remodeling and capillary angiogenesis still deserves further evaluation.

It is important to note that both adaptive changes triggered by aerobic training are essential for the improvement of brain perfusion. The arteries' wall/lumen ratio normalization, by reducing brain resistance, is positively correlated with the partial pressure decrease. Indeed, the large cerebral (and pial) arteries carry a significant amount of brain resistance (Pires et al., 2013), being important regulators of cerebral blood flow and contributors of the total peripheral resistance. On the other hand, the increased PVN and NTS capillary volume is negative correlated with reduced basal CBF. Notice that the association of both training effects (reduced brain resistance and increased capillary network) shall cause excessive local perfusion at resting condition, which is offset by active arteries/arterioles vasoconstriction that reduces basal CBF. This mechanism is specific for the WKY-T group that showed a significant reduction in basal CBF at rest, indicative of increased upstream resistance. Sustained vasoconstriction could trigger the local remodeling in some arteries. Indeed, the middle cerebral artery exhibited an increased wall/lumen ratio in WKY-T when compared with WKY-S, an indirect index for the presence of a mechanism to overcome the occurrence of overperfusion at rest. In contrast, only a small not significant reduction was observed in the SHR-T, since hypertensive rats already exhibited reduced baseline CBF when compared with age-matched controls.

In SHR and hypertensive humans, it was previously suggested that brainstem hypoperfusion by the narrowing of the vertebrobasilar system triggers sympathetic overdrive and maintains elevated pressure (Cates et al., 2012). Notice that training-induced outward remodeling of brain arteries and the increased capillary density within preautonomic areas are accompanied by a significant decrease in sympathetic vasomotor activity and reduction of both pressure levels and pressure variability, important factors to reduce end-organ damage in hypertensive subjects. Our results also showed that training-induced adaptive changes in SHR-T are associated with improved autonomic control of the heart (reduced sympathovagal balance) and resting bradycardia. WKY-T rats responded to aerobic training with angiogenesis within the NTS and PVN showing an improvement of spontaneous baroreflex sensitivity and resting bradycardia.

In conclusion, our data confirmed the deleterious remodeling of 3 important cerebral arteries and reduced CBF in chronic hypertension. Moreover, in contrast with previous findings in the peripheral tissues and cerebral cortex, we showed that hypertension is not accompanied by capillary rarefaction within important brain nuclei controlling the autonomic function. We also documented in the trained SHR an intense angiogenesis within these areas associated with arteries wall/lumen ratio normalization. These are crucial protective adjustments to reduce brain resistance and maintain local perfusion at basal condition, increasing it during exercise when vasodilation occurs.

## Data Availability Statement

The raw data supporting the conclusions of this article will be made available by the authors, without undue reservation.

## Ethics Statement

The animal study was reviewed and approved by Institutional Animal Care and Use Committee of the University of São Paulo (CEUA protocol 09/2019).

## Author Contributions

MJ participated in the research design, performed the experiments, analyzed, and revised data. AC performed the experiments, analyzed, and revised data. LM conceived, designed, coordinated the research, wrote, and revised the manuscript. All authors read and approved the final version.

## Funding

This study was supported by Fundação do Amparo à Pesquisa do Estado de São Paulo (Fapesp, research grant 2018/14544-6), Coordenação de Aperfeiçoamento de Pessoal de Nivel Superior (CAPES-Finance Code 001 – fellowship to MJ), and Conselho Nacional de Desenvolvimento Científico e Tecnológico (CNPq, grant 304070/2019-0, research Fellow to LM).

## Conflict of Interest

The authors declare that the research was conducted in the absence of any commercial or financial relationships that could be construed as a potential conflict of interest.

## Publisher's Note

All claims expressed in this article are solely those of the authors and do not necessarily represent those of their affiliated organizations, or those of the publisher, the editors and the reviewers. Any product that may be evaluated in this article, or claim that may be made by its manufacturer, is not guaranteed or endorsed by the publisher.
