# Supplementary material for: Perfusion of Brain Preautonomic Areas in Hypertension: Compensatory Absence of Capillary Rarefaction and Protective Effects of Exercise Training
Source: Front Physiol. 2021 Dec 16;12:773415. doi: 10.3389/fphys.2021.773415 (PMC8716837; doi:10.3389/fphys.2021.773415)
Supplement: Supplementary file 1 [file Table_1.docx]

**SUPPLEMENTARY MATERIAL**

**Supplementary Figure 1 Caption**. Comparisons made by Factorial Two-way ANOVA PVN*vm*: *group* F(1,12)=12.15, P=0.005, *condition* F(1,12)=130.4, P<0.001, *interaction* F(1,12)=2.35, P=0.151; PVN*mg*: *group* F(1,12)=1.20, P=0.295, *condition* F(1,12)=120.0, P<0.001, *interaction* F(1,12)=3.33, P=0.093; PVN*post*: *group* F(1,12)=6.04, P=0.030, *condition* F(1,12)=2.93, P=0.113, *interaction* F(1,12)=0.21, P=0.652.

**Supplementary Figure 2 Caption.** Comparisons made by Factorial Two-way ANOVA NTS*comm*: *group* F(1,12)=16.07, P=0.002, *condition* F(1,12)=19.06, P=0.001, *interaction* F(1,12)=0.39, P=0.545; NTS*int*: *group* F(1,12)=16.48, P=0.002, *condition* F(1,12)=16.08, P=0.002, *interaction* F(1,12)=0.79, P=0.390; NTS*med*: *group* F(1,12)=5.76, P=0.034, *condition* F(1,12)=12.17, P=0.005, *interaction* F(1,12)=13.09, P=0.004.

**Supplementary Figure 3 Caption.** Comparisons made by Factorial Two-way ANOVA BA: *group* F(1,11)=19.90, P=0.001, *condition* F(1,11)=5.69, P=0.036, *interaction* F(1,11)=4.31, P=0.062; PCA: *group* F(1,11)=1.92, P=0.193, *condition* F(1,11)=0.73, P=0.412, *interaction* F(1,11)=29.58, P<0.001; MCA: *group* F(1,11)=33.42, P<0.001, *condition* F(1,11)=15.18, P=0.003, *interaction* F(1,11)=42.92, P<0.001.

**Supplementary Figure 4 Caption.** Comparisons made by Factorial Two-way ANOVA. Basal CBF: *group* F(1,20)=27.25, P<0.001, *condition* F(1,20)=13.32, P=0.002, *interaction* F(1,20)=5.73, P=0.027; Delta CBF: *group* F(1,20)=1.04, P=0.321, *condition* F(1,20)=9.13, P=0.007, *interaction* F(1,20)=0.04, P=0.846.

**Supplementary Table 1 Caption.** Comparisons made by Factorial Two-way ANOVA. Gain: *group* F(1,54)=2.03, P=0.160, *condition* F(1,54)=184.2, P<0.001, *interaction* F(1.54)=0.23, P=0.637; SAP: *group* F(1,50)=314.8, P<0.001, *condition* F(1,50)=2.31, P=0.135, *interaction* F(1,50)=0.03, P=0.867; DAP: *group* F(1,50)=214.6, P<0.001, *condition* F(1,50)=14.19, P<0.001, *interaction* F(1,50)=1.58, P=0.215; MAP: *group* F(1,50)=191.0, P<0.001, *condition* F(1,50)=12.50, P<0.001, *interaction* F(1,50)=5.06, P=0.030; ΔP: *group* F(1,50)=50.63, P<0.001, *condition* F(1,50)=4.38, P=0.042, *interaction* F(1,50)=0.18, P=0.677; HR: *group* F(1,50)=87.16, P<0.001, *condition* F(1,50)=42.42, P<0.001, *interaction* F(1,50)=3.82, P=0.056; SAP variability: *group* F(1,17)=22.42, P<0.001, *condition* F(1,17)=8.14, P=0.011, *interaction* F(1,17)=6.43, P=0.021; LF-SAP: *group* F(1,17)=19.56, P<0.001, *condition* F(1,17)=3.95, P=0.053, *interaction* F(1,17)=3.06, P=0.098; PI variability: *group* F(1,17)=2.03, P=0.172, *condition* F(1,17)=1.56, P=0.229, *interaction* F(1,17)=1.12, P=0.304; LF/HF ratio: *group* F(1,17)=5.56, P=0.031, *condition* F(1,17)=3.06, P=0.098, *interaction* F(1,17)=4.22, P=0.056; αLF: *group* F(1,17)=27.12, P<0.001, *condition* F(1,17)=11.93, P=0.003, *interaction* F(1,17)=1.33, P=0.266; αHF: *group* F(1,17)=10.63, P=0.005, *condition* F(1,17)=17.92, P<0.001, *interaction* F(1,17)=1.89, P=0.188.

**Supplementary Table 2 Caption.** Comparisons made by Factorial Two-way ANOVA. PVN*vm*: *group* F(1,12)=7.86, P=0.016, *condition* F(1,12)=46.88, P<0.001, *interaction* F(1,12)=0.41, P=0.534; PVN*mg*: *group* F(1,12)=0.19, P=0.675, *condition* F(1,12)=74.02, P<0.001, *interaction* F(1,12)=3.47, P=0.087; PVN*post*: *group* F(1,12)=7.93, P=0.015, *condition* F(1,12)=4.25, P=0.061, *interaction* F(1,12)=0.43, P=0.523; NTS*comm*: *group* F(1,12)=4.48, P=0.050, *condition* F(1,12)=8.52, P=0.013, *interaction* F(1,12)=0.54, P=0.479; NTS*int*: *group* F(1,12)=9.61, P=0.009, *condition* F(1,12)=10.39, P=0.007, *interaction* F(1,12)=0.51, P=0.487; NTS*med*: *group* F(1,12)=0.12, P=0.741, *condition* F(1,12)=3.92, P=0.071, *interaction* F(1,12)=4.86, P=0.048.

**Supplementary Table 3 Caption.** Comparisons made by Factorial Two-way ANOVA. ***Basilar Artery*** ID: *group* F(1,11)=0.80, P=0.391, *condition* F(1,11)=6.56, P=0.027, *interaction* F(1,11)=4.86, P=0.049; OD: *group* F(1,11)=0.04, P=0.842, *condition* F(1,11)=5.70, P=0.036, *interaction* F(1,11)=5.70, P=0.036; δ: *group* F(1,11)=16.92, P=0.002, *condition* F(1,11)=2.71, P=0.128, *interaction* F(1,11)=0.68, P=0.428; CSA: *group* F(1,11)=28.94, P<0.001, *condition* F(1,11)=0.05, P=0.823, *interaction* F(1,11)=2.25, P=0.162; ***Posterior Cerebral Artery*** ID: *group* F(1,11)=13.47, P=0.004, *condition* F(1,11)=0.01, P=0.923, *interaction* F(1,11)=7.17, P=0.022; OD: *group* F(1,11)=13.05, P=0.004, *condition* F(1,11)=0.00, P>0.999, *interaction* F(1,11)=0.66, P=0.434; δ: *group* F(1,11)=0.36, P=0.563, *condition* F(1,11)=0.00, P>0.999, *interaction* F(1,11)=28.86, P<0.001; CSA: *group* F(1,11)=12.72, P=0.004, *condition* F(1,11)=0.57, P=0.465, *interaction* F(1,11)=7.02, P=0.023; ***Middle Cerebral Artery*** ID: *group* F(1,11)=0.02, P=0.886, *condition* F(1,11)=4.84, P=0.050, *interaction* F(1,11)=43.55, P<0.001; OD: *group* F(1,11)=1.51, P=0.245, *condition* F(1,11)=4.30, P=0.062, *interaction* F(1,11)=62.69, P<0.001; δ: *group* F(1,11)=18.46, P=0.001, *condition* F(1,11)=1.60, P=0.233, *interaction* F(1,11)=0.58, P=0.464; CSA: *group* F(1,11)=4.61, P=0.055, *condition* F(1,11)=2.88, P=0.118, *interaction* F(1,11)=31.05, P<0.001.
